# Prevalence and Antifungal Susceptibility Profile of Oral *Candida* spp. Isolates from a Hospital in Slovakia

**DOI:** 10.3390/medicina58050576

**Published:** 2022-04-22

**Authors:** Lucia Černáková, Anna Líšková, Libuša Lengyelová, Célia F. Rodrigues

**Affiliations:** 1 Department of Microbiology and Virology, Faculty of Natural Sciences, Comenius University in Bratislava, 842 15 Bratislava, Slovakia; 2 Department of Clinical Microbiology, Nitra Faculty Hospital, 950 01 Nitra, Slovakia; aliskova@gmail.com; 3 Department of Botany and Genetics, Faculty of Natural Sciences and Informatics, Constantine the Philosopher University in Nitra, 949 01 Nitra, Slovakia; llengyelova@ukf.sk; 4 TOXRUN—Toxicology Research Unit, Cooperativa de Ensino Superior Politécnico e Universitário—CESPU, 4585-116 Gandra, Portugal; 5 LEPABE—Laboratory for Process Engineering, Environment, Biotechnology and Energy, Faculty of Engineering, University of Porto, 4200-465 Porto, Portugal

**Keywords:** *Candida albicans*, NCAC, *Candida* spp., resistance, antifungals, oral infection

## Abstract

Oral fungal infections are a worldwide healthcare problem. Although *Candida albicans* is still the most common yeast involved in the infections of oral cavity, non-*Candida* *albicans* *Candida* species (NCACs) have been highly related to these infections, particularly in older, immunosuppressed or patients with long exposure to antimicrobial drugs. The goal of this work was to perform a quick epidemiological and mycological study on the oral samples collected from a laboratory of a hospital in Slovakia, for 60 days. The samples’ identification was performed by Germ-tube formation test, CHROMID^®^ *Candida,* Auxacolor 2, ID 32C automated method, and the antifungal susceptibility testing determined by E-test^®^. Results confirm that comparing with bacteria, yeasts still occur in the lower number, but there is a high rate of antifungal resistance (81.6%)—to, at least one drug—among the collected samples, particularly to azoles and 5′-FC, which is clinically noteworthy.

## 1. Introduction

Oral infections are a heavy economic burden, a worldwide priority, and urgent problem, that can lead to pain, tooth loss, disfigurement, and death [1,2,3]. Since the 1980s, the studies on oral infections by *Candida albicans* have increased due to the escalation in HIV infection and the acquired immune deficiency syndrome (AIDS) epidemic [4]. Recent global estimates indicate an incidence of 2 million cases of oral candidiasis (i.e. thrush) annually, which, with skin, hair, and nail fungal infections, is the highest among fungi diseases [5]. In fact, oral candidiasis (OC) has been described throughout human history, since the times of Hippocrates (460–370 bC, “a disease of the diseased”), mirroring the opportunistic pathogenic nature of *Candida* spp. [6]. Later, in 1838, the infection was first described as directly linked specifically to *Candida albicans*, by pediatrician Francois Veilleux [7]. However, recently, non-*Candida albicans Candida* species (NCACs), such as *Candida glabrata, Candida tropicalis*, and *Candida krusei* have been highly related to these infections [8,9], colonizing mostly (but not only) older patients [10]. Oral candidiasis is diagnosed through differential patterns of mucosal changes (e.g., erythematous, pseudomembranous, and curd-like plaques—biofilms) [11,12].

Like other fungal infections, OC occurs as a consequence of other health problems, such as cancer, HIV, tobacco smoking, denture wearing, chemotherapy, and corticosteroid therapies, linked to immunosuppression states. The risk factors for oral candida infection are complex, but these factors clearly influence oral *Candida* sp. carriage and the upsurge of oral candidiasis [13,14,15,16,17,18,19]. For example, in patients with diabetes, there is a higher *Candida* spp. colonization rate than on non-diabetic subjects (60–80% vs. 27%) [20]. It is, however, important to note that the colonization rate does not always lead to disease, but it is a run-up to infection when host immunity is compromised [21,22]. Hence, an early precise diagnosis can grant a quick and efficient antifungal therapy [23]. Conversely, this is repeatedly overdue or unavailable leading to death or serious chronic illness, nonetheless, they are still overlooked by public health authorities while most mortalities from these disorders are, in fact, preventable [5,22].

Additionally, up-to-date reports indicate an escalation of antimicrobial resistance in oral microbial species (oral microbiome) [24,25,26,27,28], and mounting evidence also supports a major role in systemic extra-oral infections and inflammation (e.g., cardiovascular, neurological) [29,30,31]. Not differently, it has been reported that, particularly in older adults, the oral colonization by *Candida* spp. increases and is related to other morbidities (e.g., Crohn’s disease) [32,33,34,35]. This is mostly true for NCACs, which present a reduced response to fluconazole, raising the risk of recalcitrant local and disseminated candidiasis [36,37].

To our knowledge, this is the first retrospective re-examination study, evaluating and characterizing the clinical isolates of *Candida* spp. collected in the Department of Clinical Microbiology, Nitra Faculty Hospital in Slovakia. The collection of the samples was performed over a period of 60 days in 2019, relating them to wards, gender, ages, and antifungal susceptibility profiles.

## 2. Materials and Methods

### 2.1. Collection and Identification of the Clinical Isolates

Samples and clinical data of patients were collected between April and May 2019, from the laboratory of the Department of Clinical Microbiology, from several wards of the Nitra Faculty Hospital in Slovakia. Various tests were performed to confirm the identification of the samples.

#### 2.1.1. Germ-Tube Formation Test, CHROMID^®^ Candida, Auxacolor 2 (Bio-Rad®) Test

All clinical isolates were first plated on Sabouraud dextrose agar (SDA, Thermo Fisher Oxoid, UK) for 24 h at 37 °C. Afterwards, germ-tube test was performed to differentiate between *Candida* spp. Colonies of the cultivated yeasts were suspended in sterile saline and adjusted to the density of McFarland 2. For the morphological examination, human serum was used. Two drops of the yeast suspension were applied to wells of microtiter plates with 30 µL of human serum. After incubation for 2–3 h at 37 °C, the number of the germ tube and the total number of the cells was calculated in Bürker counting chamber. Production of chlamydospores, blastospores, true hyphae, and pseudohyphae was compared.

Parallelly, CHROMID^®^ *Candida* (bioMérieux^®^, Craponne, France) was used for presumptive identification of *Candida strains*, based on the pigmentation of the developing colonies due to different enzyme activity. The results were interpreted after 48 h of incubation at 30 °C, according to colony’s color and manufacturer’s instructions. Subsequently, for identification of the medically important isolated yeasts, the colorimetric sugar assimilation test Auxacolor 2 (Bio-Rad^®^, Hercules, CA, USA) served for accurate identification. The method was applied as described in the kit’s guideline and results were then evaluated.

#### 2.1.2. The ID 32C Automated Method

The cultures were also analyzed macroscopically and microscopically and confirmed by the ID 32C automated method—ATB Expression (bioMérieux^®^, Craponne, France). For this, a single-use disposable plastic strip was used; 32 wells contain substrates for 29 assimilation tests (carbohydrates, organic acids, and amino acids), one susceptibility test (cycloheximide), one colorimetric test (esculin), and negative control. Testing was performed according to the guideline of the manufacturer.

### 2.2. Antifungal Susceptibility Testing

For all the isolates, the antifungal susceptibility for fluconazole (Flu), voriconazole (Vcz), 5′-fluorouracil (5FC), and amphotericin B (AmB) (i.e., the most common antifungal drugs used to treat oral infections [38]) was determined by the agar-based E-test^®^ (bioMérieux, Craponne, France), with RPMI-1640, 8.4 g/L (Roswell Park Memorial Institute; Sigma Chemical Co., St. Louis, MO, USA), 2% glucose (Thermo Fisher Oxoid, UK) and 1.5% agar (NEOGEN, Lansing, MI, USA) buffered with 0.165 M morpholinepropanesulfonic acid buffer (MOPS; pH = 7; Sigma Chemical Co., St. Louis, MO, USA). The plates were inoculated by dipping a sterile swab into the inoculum suspension adjusted to the turbidity of a 0.5 McFarland standard and streaking it across the surface of the agar in four directions. The plates were dried at ambient temperature for 15 min before applying the E-test^®^ strips. The minimum inhibition concentrations (MIC) endpoints were determined after 24 and 48 h of incubation at 35 °C. The MIC was read for Amphotericin B (AmB), as the drug concentration that zone determined the point of complete inhibition (100%), and for Voriconazole (Vcz), as required by the method [39].

### 2.3. Software and Statistical Analysis

The analysis was performed using GraphPad Prim v.7 (GraphPad Software, CA, USA). All experimental data are presented as mean ± standard error of the mean (SEM).

## 3. Results

**Figure 1:**
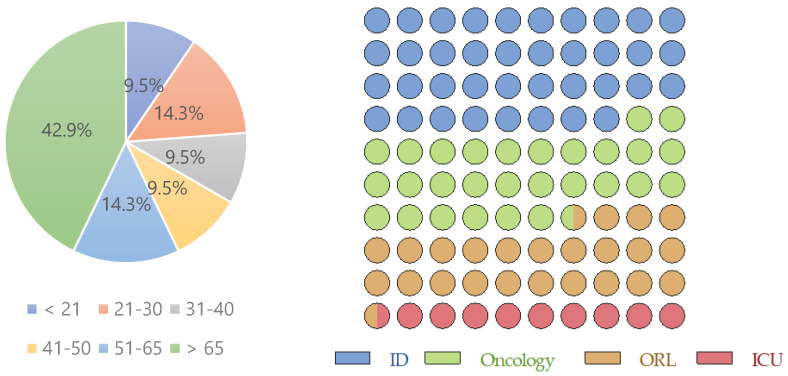
Distribution and percentage of ages and samples collected from specific hospital wards. (*n* = 21; Female: *n* = 8, 38.1%; Male: *n* = 13, 61.9%; ID—infectious diseases; ORL—otorhinolaryngology” (ORL) and ICU—intensive care unit).

Here, we evaluated the rate of antifungal resistance of *Candida* oral samples in our laboratory. For this, we collected oral isolates from different wards of the Department of Clinical Microbiology, Nitra Faculty Hospital in Slovakia, for 60 days. Among the 62 oral samples collected, 21 isolates were yeasts of *Candida* genus, from four different hospital wards: Infectious diseases (ID), Oncology, Otorhinolaryngology (ORL), and Intensive Care Unit (ICU). Not surprisingly, most patients came from ID ward, followed by Oncology and ORL (Figure 1, Table 1 and Table 2). The average age of the cohort was 51.9 ± 19.7 years (female: 48.6 ± 21.0 and male: 52.3 ± 20.4). These results indicate that the oral *Candida* spp. prevalence (in this hospital and period) was higher among males than among females (61.9% vs. 38.1%). Men were also older than females, who came mostly from ID ward (50.0%), followed by Oncology (25.0%), ORL, and ICU (both with 12.5%). On the other side, male subjects came equally from ID, Oncology and ORL (30.7% each), followed by ICU (7.7%).

It is relevant to note that from all the identified samples of the hospital, the ratio of yeasts to bacteria is, in general, about 1/5. Nevertheless, taking into consideration only samples from the oral cavity, there is the prevalence of the yeast strains. Furthermore, of the collected *Candida* spp., the most prevalent was *C. albicans* (61.9%, *n* = 13), followed by *C. krusei* (14.3%, *n* = 3), *C. valida* (9.5%, *n* = 2), and *C. glabrata*, *C. tropicalis*, and *C. intermedia* (all: 4.8%, *n* = 1) (Table 3).

After performing the antifungal susceptibility tests, all isolates were sensible to AmB, but several (*n* = 11) had resistance to 5FC (52.4%), to Flu (28.5%, *n* = 6) and Vcz (intermediate profile: 0.95%, *n* = 2). This means that 81.6% of the collected strains had some resistance to one or more antifungal drugs, which is clinically noteworthy.

## 4. Discussion

Even if *Candida* spp. is a member of the oral microbiota, in some circumstances, the increase of *Candida* spp. population can lead to dysbiosis, easily originating oral candidiasis. Among the more than 350 heterogeneous species of *Candida*, *C. albicans* is the most prevalent in human OC [40]. For both *C. albicans* and NCACs, the transition from a harmless commensal to a pathogen is complex and poorly understood, although it seems to include different expression of virulence factors [6,41]. Moreover, it is known that NCACs have been progressively encountered and recognized as important agents of human oral infections [6,40,42,43,44,45]. This has also been aggravated by COVID-19 pandemic since an increase in oral candidiasis has been seen in these patients [46].

In this work, we performed a characterization of the antifungal profile of *Candida* oral samples from Nitra Faculty Hospital in Slovakia, collected for 60 days. It is pertinent to note that, although studies similar as this one (with short samples) have been published related to other countries (discussed in the next lines), we still do not know the status in Slovakia related to antifungal susceptibility/resistance of *Candida* clinical isolates, which made this study comprehensively relevant. The 21 *Candida* isolates were from several wards: ID, Oncology, ORL, and ICU, and most patients were from ID and Oncology wards (Figure 1, Table 2). The oral *Candida* spp. prevalence was higher among males when comparing with females. Besides, men were older than females. In fact, Naidu et al. documented similar results, with male predominance. It was indicated that, among HIV patients with oral lesions, including OC, 44% were male and 22% were female, aged between 20 and 55 years [47]. Even so, different gender balances also have occurred related to candidiasis in general [48,49,50,51].

With no surprises, *C. albicans* was the most common species (61.9%, *n* = 13), followed by *C. krusei* (14.3%), *C. valida* (9.5%) and *C. glabrata*, *C. tropicalis*, and *C. intermedia* (all: 4.8%) (Table 3). Similar epidemiological results have been reported. Arlsan and co-workers (Turkey) found 12 different genotypes and compared the virulence factors of several oral *Candida* spp. isolated from individuals with and without caries. The most isolated species was *C. albicans*, and different genotypes exhibited different virulence activities [52]. Correspondingly, Aitken-Saavedra et al. (Chile) revealed that 66% of the isolated yeasts were *C. albicans*, followed by *C. glabrata* (20.7%) [53]. In fact, in the last years, NCAC have been more identified in oral infections. In our case, around 27% of the isolates were NCAC, with *C. krusei* showing to be the most common (Table 3). In 2015, Indira et al. (India) [54] also reported that between 35 and 49% of oral candidiasis were related to *C. krusei*, which was, in fact, the most common *Candida* sp. isolated in people living with HIV with diabetes (50%). Other similar results (China and Iran) corroborate our results, with this species being the most or one of the most NCAC isolated (both in single and multi-fungal cultures) [55,56,57].

Regarding the antifungal susceptibility tests, half isolates presented resistance to 5FC, and almost 29% has resistance to Flu, hence almost 82% of the collected samples showed resistance to, at least, one antifungal class, which is clinically remarkable. All isolates were sensible to AmB and two samples had intermediate profile to Vcz. Previous research showed the effectiveness of Vcz and AmB *Candida* spp. isolates [25,26,55,58,59,60,61,62] (Portugal, Brazil, Korea, US, Japan), but also of both AmB and Flu (China) [57], as seen in this work. A recent study involving immunosuppressed patients (with diabetes mellitus), showed a predominance of *C. albicans*, but among denture wearers, *C. glabrata* was prevalent. Importantly, this report indicated that ketoconazole, Flu, and itraconazole were effective against the isolated *Candida* spp. (India) [63]. Correspondingly, Premkumar et al. (India) stated that, although *C. albicans* was the most predominantly isolated species, NCACs were also observed (e.g., *C. dubliniensis*, *C. tropicalis*, and *C. parapsilosis*) [64]. The authors indicated a variable resistance toward AmB and Flu [64]. Finally, it has been suggested that, in patients with oral infections and dentures, chlorhexidine (CHX) is an effective anti-*Candida* spp. CHX showed to have a good antiseptic effect on *Candida* sp. by killing it and preventing new adhesion [65]. This has also been showed in a recent work, with CHX being placed in a medical device [66] and in patients with long oral carriage of *Candida* spp. [61]. Hence, in the future, it might be important to start evaluating the susceptibility of this drug in *Candida* spp. oral isolates.

## 5. Conclusions

This preliminary work shows that, comparing with bacteria, yeasts are still isolated in a lower number in hospitalized patients in Slovakia. Nonetheless, and critically, there is a high rate of antifungal resistance to, at least one, drug (particularly to azoles and 5′-FC—around 82%), which is an important clinical finding.

It is, however, essential to state that the low number of samples is a limitation of this study, and more studies should be performed in the future to support our conclusions. Although echinocandins are not used to treat OC, adding information on the echinocandin could also improve the assumptions. Moreover, the fact that VITEK or mass spectrometry was not used could also be considered a limitation, since misidentification of species could have occurred. Nonetheless, using more than one identification method for each sample (germ-tube formation test, CHROMID^®^ Candida, Auxacolor 2 Bio-Rad^®^ test, ID 32C automated method—ATB Expression bioMérieux^®^), provided high confidence in the identifications.

Taking into consideration the increasing number of *Candida* spp. oral strains, at a significant rate of drug resistance, the identification of effective alternative therapies (such as photodynamic therapy or natural compounds) to the current antifungal agents is essential.

## Figures and Tables

**Table 1:** Gender and age distribution of the collected samples.

	<21	21–30	31–40	41–50	51–65	>65
Female	1 (4.8%)	1 (4.8%)	1 (4.8%)	1 (4.8%)	1 (4.8%)	3 (14.3%)
Male	1 (4.8%)	2 (9.5%)	1 (4.8%)	1 (4.8%)	2 (9.5%)	6 (28.6%)
Total	2 (9.5%)	3 (14.3%)	2 (9.5%)	2 (9.5%)	3 (14.3%)	9 (42.9%)

**Table 2:** Gender and ward distribution of the collected samples.

	ID	Oncology	ORL	ICU
Female	4 (19%)	2 (9.5%)	1 (4.8%)	1 (4.8%)
Male	4 (19%)	4 (19%)	4 (19%)	1 (4.8%)
Total	8 (38.1%)	6 (28.6%)	5 (23.8%)	2 (9.5%)

ID: infectious diseases; ORL: otorhinolaryngology; ICU: intensive care unit.

**Table 3:** Total number and percentage, identification of the collected oral *Candida* spp. isolates.

*Candida* spp.	*n*	%	Susceptibility Profile			
			Flu	Vcz	5FC	AmB
*C. albicans*	13	61.9	S	S	**R**	S
			S	S	S	S
			S	S	**R**	S
			S	S	**R**	S
			S	S	S	S
			S	S	**R**	S
			S	S	S	S
			S	S	**R**	S
			S	S	S	S
			S	S	**R**	S
			S	S	**R**	S
			S	S	S	S
			S	S	S	S
*C. krusei*	3	14.3	**R**	S	**R**	S
			**R**	S	**R**	S
			**R**	S	**S**	S
*C. valida*	2	9.5	**R**	**I**	S	S
			**R**	**I**	S	S
*C. tropicalis*	1	4.8	S	S	**R**	S
*C. glabrata*	1	4.8	S	S	S	S
*C. intermedia*	1	4.8	**R**	S	**R**	S

S: sensible; R: resistant; I: intermediate (**Bold**: intermediate and resistance to the antifungal drug).
